# Changes in Structure and Functioning of Protist (Testate Amoebae) Communities Due to Conversion of Lowland Rainforest into Rubber and Oil Palm Plantations

**DOI:** 10.1371/journal.pone.0160179

**Published:** 2016-07-27

**Authors:** Valentyna Krashevska, Bernhard Klarner, Rahayu Widyastuti, Mark Maraun, Stefan Scheu

**Affiliations:** 1 Georg August University Göttingen, J.F. Blumenbach Institute of Zoology and Anthropology, Göttingen, Germany; 2 Institut Pertanian Bogor—IPB, Department of Soil Sciences and Land Resources, Damarga Campus, Bogor, Indonesia; Stockholm University, SWEDEN

## Abstract

Large areas of tropical rainforest are being converted to agricultural and plantation land uses, but little is known of biodiversity and ecological functioning under these replacement land uses. We investigated the effects of conversion of rainforest into jungle rubber, intensive rubber and oil palm plantations on testate amoebae, diverse and functionally important protists in litter and soil. Living testate amoebae species richness, density and biomass were all lower in replacement land uses than in rainforest, with the impact being more pronounced in litter than in soil. Similar abundances of species of high and low trophic level in rainforest suggest that trophic interactions are more balanced, with a high number of functionally redundant species, than in rubber and oil palm. In contrast, plantations had a low density of high trophic level species indicating losses of functions. This was particularly so in oil palm plantations. In addition, the relative density of species with siliceous shells was >50% lower in the litter layer of oil palm and rubber compared to rainforest and jungle rubber. This difference suggests that rainforest conversion changes biogenic silicon pools and increases silicon losses. Overall, the lower species richness, density and biomass in plantations than in rainforest, and the changes in the functional composition of the testate amoebae community, indicate detrimental effects of rainforest conversion on the structure and functioning of microbial food webs.

## Introduction

The biodiversity of natural ecosystems is indispensable for providing ecosystem functions, but is threatened by anthropogenic activities such as the conversion of forests into agricultural production systems. Tropical forests have very high biodiversity so there is particular and increasing concern about their conversion into agricultural and plantation systems [1,2]. Nevertheless, tropical forest conversion is increasing rapidly [3], particularly in South East Asia. On Sumatra, for example, 12 million ha of forest have been converted over the past 30 years [4], predominantly into oil palm and rubber plantations [5]. However, despite the large-scale conversion of rainforests worldwide, little is known about the biodiversity and ecological functioning of the land uses replacing lowland rainforest [6]. This applies, in particular, to the functioning of the highly diverse belowground system [7].

The belowground decomposer food web systems are composed of microorganisms, micro-, meso- and macrofauna, interacting with each other and the environment in a complex network. These interactions drive the major functions of terrestrial ecosystems, such as nutrient mineralization and plant productivity. A particularly wide spectrum of these biochemical transformations are carried out by saprotrophic soil microorganisms. Fungi and bacteria are thus particularly influential and constitute the base of soil food webs [8]. Bacteria are grazed by other organisms, most importantly soil protists and nematodes [9–11]. By grazing on bacteria, protists release nutrients fixed in bacterial biomass, thereby increasing mineralization of carbon and nitrogen [12]. These are fundamental processes and, therefore, changes in the numbers and functional identities of protists may have major effects on ecosystem functioning. Nevertheless, in general, there is little study of the conversion of tropical lowland rainforest to other land uses and the associated changes in soil biodiversity and soil biota functioning. The effects of rainforest conversion on protists are, in fact, virtually unknown despite their outstanding importance in trophic interactions with microorganisms and their role in nutrient cycling.

We investigated changes in protists density, biomass and species number with rainforest conversion allowing to identify changes in community functioning and the factors responsible for these changes. Testate amoebae are among the main protist groups in acidic soils including those of typical lowland tropical rainforests. Testate amoebae are sensitive to disturbance and therefore changes in their numbers and community structure reflect changes in abiotic and biotic factors associated with rainforest conversion [13–16]. Furthermore, testate amoebae have a variety of feeding types and trophic positions [17–19]. Therefore, differential changes in the abundance of feeding types might provide insight into structural changes of soil microbial food webs with rainforest conversion. However, methods for classifying testate amoebae into trophic groups are poorly developed. Based on our own analyses, size measurements and literature data, we grouped testate amoebae into high and low trophic level taxa. Testate amoebae preferably feeding on bacteria, fungi or microscopic algae e.g. [20–23] were grouped as low and species preferably feeding on single and multicellular eukaryotes (e.g., other protists, nematodes and rotifers [24,25,20]) were grouped as high trophic level. Additionally, testate amoebae are shelled protists and the chemistry of their shells is closely linked to soil chemistry; e.g. taxa with siliceous shells have been shown to contribute significantly to the terrestrial silicon cycle [26,27]. Anthropogenic disturbances such as rainforest conversion may result in redistribution of silicon in soils and increased silicon loss [28,29]. Moreover, silicon loss may impact carbon fluxes since the biogenic silicon cycle is closely linked to the carbon cycle [28,30]. Furthermore, silicon losses might affect plants directly because silicon plays a major role in plant performance [31]. Therefore, differential changes in the abundance of species with siliceous shells might reveal the impact of forest conversion on the biogenic silicon cycle.

We expected that (1) the impact of rainforest conversion on testate amoebae increases with management intensity, i.e. from rainforest to jungle rubber to rubber to oil palm plantations, (2) the impact of rainforest conversion on testate amoebae differs between litter and soil being more pronounced in litter as litter is more heavily exposed to environmental variation than deeper soil layers [32], (3) the density of testate amoebae of low trophic level increases (and that of high trophic level decreases) with management intensity as high trophic level taxa are more sensitive to disturbance [17,33], and (4) the density of testate amoebae with siliceous shells decreases with management intensity as they are sensitive to anthropogenic change [13–16,27,34]. In the tropical lowland of Sumatra, Indonesia, we quantified the impact of rainforest conversion into other land uses: jungle rubber, rubber plantations and oil palm plantations. We investigated the density, diversity and biomass of living testate amoebae in the litter layer and in upper mineral soil. Furthermore, we investigated the impact of rainforest conversion on testate amoebae community structure, including their trophic positions and the proportion of species with siliceous shells. To identify factors responsible for changes in testate amoebae community structure we measured a wide spectrum of abiotic and biotic environmental factors that differ between rainforest and other land uses [32].

## Materials and Methods

### Study sites and sampling

The study took place in the tropical lowlands of Jambi Province in southeast Sumatra, Indonesia. The climate is generally tropical humid with a mean annual air temperature of 26.7 ± 0.1°C and a mean annual precipitation of 2235 ± 385 mm, with the rainy season lasting from October to April [35]. There were two localities, Bukit Duabelas (2° 0' 57" S, 102° 45’ 12" E) and Harapan (1° 55' 40" S, 103° 15' 33" E) [36], and at each we studied four conversions of rainforest to other land uses: rainforest, jungle rubber, rubber plantations and oil palm plantations. Each land use was replicated four times in each locality, resulting in 32 sampling sites. Rainforest used as reference system was represented by old growth secondary rainforest. Jungle rubber represents a non-intensive rubber (*Hevea brasiliensis*) agroforestry system with interspersed native tree species; the age of the rubber trees was on average 29 years [37]. Rubber plantations represented rubber monocultures of an average age of 13 years [37]. Oil palm (*Elaeis guineensis*) plantations represented oil palm monocultures of an average age of 14 years [37]. Rubber and oil palm monocultures were managed by smallholders and received NPK, urea and potassium chloride as fertilizers as well as lime [36]. Jungle rubber is typically little fertilized. During our study period in 2013 only oil palm plantations were fertilized [36]. In Harapan (with loam Acrisol soil) they were fertilized twice. First between October and March (rainy season) and second between April and September (dry season). In Bukit Duabelas (with clay Acrisol soil) fertilizers were added only once during the rainy season [36]. In 2013 the dry season started in mid June and lasted until end of October [35]. For more details on the study sites see [33,35–38].

Litter (L/F horizon) and upper mineral soil samples (Ah horizon, to a depth of 50 mm) were taken in October/November 2013, using a corer 50 mm in diameter. Three cores were taken from each plot in order to account for small scale spatial variation. Litter and top soil samples were pooled from each set of three cores. Seeds, twigs, roots and coarse woody debris were removed by hand. One part of each sample was taken for the analysis of testate amoebae, the other for analysing environment variables, i.e. water content, microbial biomass, pH, amount of litter, carbon and nitrogen concentration of litter, phospholipid fatty acid (PLFA) and neutral lipid fatty acid (NLFA) markers. Detailed information on these environmental variables and methods of analysis are given in [32].

### Field work permission

The study was conducted in the framework of the German—Indonesian research project ‘Ecological and socio-economic functions of tropical lowland rainforest transformation systems’ (EFForTS) and is based on the Research permits no. 332/SIP/FRP/SM/IX/2012, 389/SIP/FRP/SM/X/2013 and 145/SIP/FRP/SM/V/2013 issued by the State Ministry of Research and Technology of the Republic of Indonesia (RISTEK). Sample exportation for analysis in Germany was based on permit no. 125/KKH-5/TRP/2014 issued by Ministry of Forestry of the Republic of Indonesia.

### Testate amoebae

Litter and soil samples (1 g dry weight) were moistened for 24 h with sterile tap water to facilitate detachment of the shells of testate amoebae from the litter and soil particles. Testate amoebae were extracted by washing the samples through 500 μm mesh and then sieving the filtrate through 10 μm mesh. From the filtrate, microscopic slides were prepared and the testate amoebae examined at 200-1000x magnification. The individuals were grouped into taxonomic units based on morphological characters of the shell, pseudopodia and nucleus [39–46]. Most individuals could be assigned to named species, others were numbered as operational taxonomic units (see S1 Table). For simplicity, we use ‘species’ for all these morphologically recognisable units. We counted the number of individuals in each species and separated living cells from empty shells [16]. We then analysed the data for the living testate amoebae. The density of individuals was calculated per gram of air-dry litter or soil. The biomass for each living species was calculated by converting the volume of species into biomass using standard conversion factors [47].

We assigned each species to either low or high trophic level based on four sources of information: (1) correlations between testate amoebae and potential prey organisms present in the samples, (2) shell and aperture size of testate amoebae [18], (3) published information on their diet, e.g. [18,19,22], and (4) our own observations (S2 Table). Correlations in source of information 1 were based on Pearson correlations between the density of testate amoebae species and fatty acids (PLFA and NLFA) in litter and soil, partly taken from [32] (see S3 and S4 Tables). PLFAs i15:0, a15:0, 15:0, i16:0, 16:1ω7, 17:0, i17:0, cy17:0, 18:1ω7 and cy19:0 were taken to represent bacterial PLFAs [48] with PLFAs 16:1ω7, cy17:0 and cy19:0 representing Gram-negative (Gr-) and PLFAs i15:0, a15:0, i16:0 and i17:0 to representing Gram-positive (Gr+) bacteria [49,50]. PLFAs 18:2ω6,9, 18:3ω6 and 18:3ω3 were taken to represent fungi [48,51]. PLFAs 20:2 and 20:4ω6,9,12,15 were used as animal markers [52,53]. PLFA 20:5ω3 was used as a marker for algae [54,55]. NLFA 16:1ω5c was used as a marker for arbuscular mycorrhizal fungi [56]. Positive correlations between testate amoebae species and bacterial, fungal or algal PLFAs were taken to group species into low trophic level taxa (scoring -1). Positive correlations between testate amoebae species and PLFAs of single and multicellular eukaryotes (animal markers) were taken to group species into high trophic level taxa (scoring +1). Shell and aperture size of testate amoebae (source of information 2) was measured on microscopic slides for each living testate amoeba individual; means per species are given in S2 Table. Species with a shell size < 60 μm and aperture diameter < 15 μm are assumed to feed on small prey likely to be of low trophic level ([18], but see [24]), whereas species with shell size > 60 μm and aperture diameter > 15 μm are likely to feed on large prey presumably of higher trophic level. The ratio between aperture diameter and shell size was used to assign species to low and high trophic level with a ratio < 0.25 indicating low trophic level (scoring -1) and a ratio > 0.25 indicating high trophic level (scoring +1).

To investigate the impact of rainforest conversion on shell composition we separated testate amoebae into two groups, those with siliceous and those with non-siliceous shells (“other”; see S2 Table; [39–46]).

### Calculations and statistical analyses

The effects of forest conversion on living species number, density and biomass of testate amoebae in litter and soil were analysed using a mixed effects model (GLMM) with land use (rainforest, jungle rubber, rubber, oil palm) as fixed factors (type III sum of squares) and locality (Harapan, Bukit Duabelas) as random factor in (SAS version 9.3: SAS Institute, Cary, NC, USA). We used Tukey´s HSD to test for *post hoc* significant differences between means. Levels of significance in text and figures are indicated as ^ns^ (P>0.05), * (P<0.05), ** (P<0.01) and *** (P<0.001).

To evaluate the effects of forest conversion on testate amoebae community structure, the multivariate dataset consisting of 85 species for litter and 92 species for soil was reduced to four dimensions using non-metric multidimensional scaling (NMDS; STATISTICA 12.0 for Windows; StatSoft, Tulsa, USA). NMDS stress values indicated that these four dimensions represented most of the information in the data. These four dimensions were analysed by discriminant function analysis (DFA; STATISTICA 12.0 for Windows) to identify effects of conversion on testate amoebae community composition in litter and soil. Squared Mahalanobis distances (MD^2^) between group centroids (rainforest, jungle rubber, rubber, oil palm) and the reliability of sample classification were determined to identify significant differences in community composition of testate amoebae between land-use systems.

To assign species to high and low trophic level groups, we calculated a coefficient integrating four sources of information: (1) our correlations described above between density of testate amoebae species and bacterial, fungal, algal and animal PLFAs, (2) the ratio of aperture diameter and shell size, (3) literature based trophic grouping, and (4) personal microscopic observations of the prey of testate amoebae. For each source of information species were scored either -1 (low trophic level) or +1 (high trophic level) and the scores then summed up as the trophic group coefficient. Species with negative coefficients were assigned to the low trophic level, those with positive coefficients to the high (for details see S3 Table). We then calculated the relative density of the two trophic groups in each plot. Thereafter, we calculated the relative density of the two shell composition groups in each plot. The relative density was calculated as the number in the target group as a percentage of the total density in a plot. The relative densities were logit-transformed before analysis. The trophic groups and shell composition groups were then analysed by multivariate analysis of variance (MANOVA; STATISTICA 12.0 for Windows).

In addition, Pearson correlation coefficients were used to investigate correlations between environmental factors in litter and soil (soil temperature, water content, microbial biomass, pH, amount of litter, carbon and nitrogen concentration, phospholipid fatty acid (PLFA) and neutral lipid fatty acid (NLFA) markers; partly taken from [32]) and testate amoebae shell composition and trophic group; for details see S3 Table and S4 Table.

The relationships between testate amoebae species density (dependent variables) and abiotic and biotic environmental factors (independent variables: soil temperature, water content, microbial biomass, pH, amount of litter, carbon and nitrogen concentrations, phospholipid fatty acid (PLFA) and neutral lipid fatty acid (NLFA) markers; partly taken from [32], see S3 Table) were analysed using canonical correspondence analysis (forward selection CCA; CANOCO 5.02 [57]). CCAs were performed because the response data were compositional and the length of gradient exceeded 3.9 SD units indicating unimodal species-environment relationships [57]. Monte-Carlo tests (999 permutations) were performed to evaluate the overall significance and the significance of environmental variables and individual axes. Since the global test with all environmental variables was significant, we used forward selection to identify the most important environmental variables structuring testate amoebae communities. The forward selection procedure was stopped if a variable reached a level of significance above 0.05. Land uses (rainforest, jungle rubber, rubber, oil palm) were included as passive variables. Only species occurring in at least two replicates were included in the CCA.

## Results

### Species number

**Fig 1:**
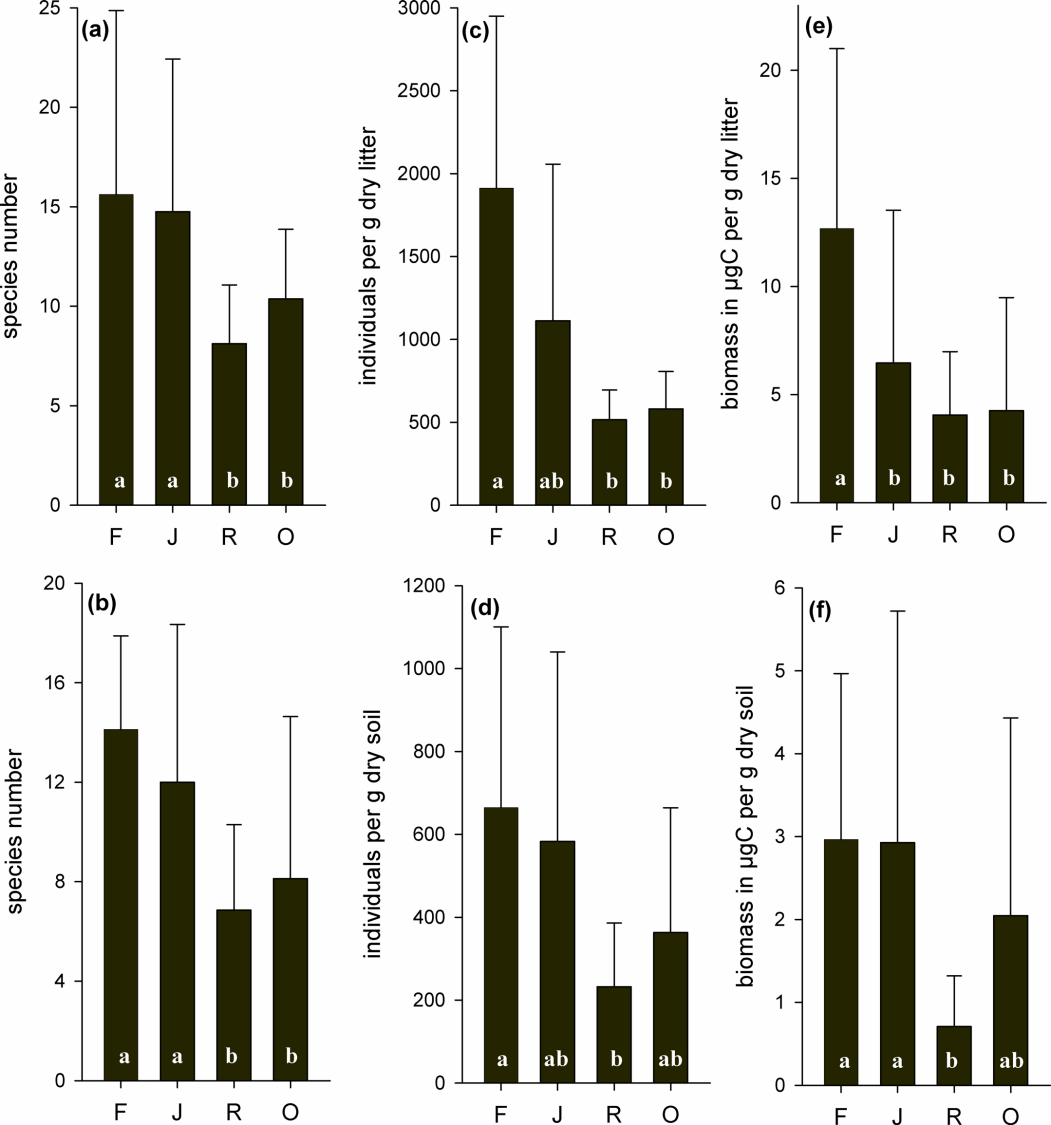
**Species number (a, b), density (c, d) and biomass (e, f) of living testate amoebae in litter (upper panel) and soil (lower panel) of four land-use systems:** rainforest (F), jungle rubber (J), rubber (R) and oil palm (O); means with SD (n = 8). Bars sharing the same letter do not differ significantly (Tukey’s HSD test, p<0.05).

There were 131 species of living testate amoebae. Of these, 85 were in litter and 92 in soil (for species list and relative abundance of the species, see S1A and S1B Table). In litter and in soil, the mean species number was similar in rainforest and jungle rubber, but 39% lower than this on average in rubber and 43% lower in oil palm (F_3,27_ = 3.23* for both; Fig 1A and 1B).

### Density

In litter and soil, density of testate amoebae varied significantly between land-use systems. In litter from jungle rubber, the density was 42% less than in rainforest; in rubber it was on average 73% lower and in oil palm 70% lower (F_3,27_ = 7.89***; Fig 1C). In soil from rubber plantations, the density of testate amoebae was 65% lower than in rainforest; and density was 12% lower in jungle rubber and 45% lower in oil palm (F_3,27_ = 3.16*; Fig 1D).

### Biomass

Biomass of testate amoebae in litter differed significantly between land-use systems. In litter from jungle rubber, the biomass was 49% less than in rainforest; in rubber it was on average 68% and in oil palm 66% lower (F_3,27_ = 3.08*; Fig 1E). In contrast to the differences found for litter, the biomass in soil was similar in rainforest and jungle rubber, lower than this in oil palm (by 30%) and lowest in rubber (by 76%; F_3,27_ = 12.8***; Fig 1F).

### Community structure

**Fig 2:**
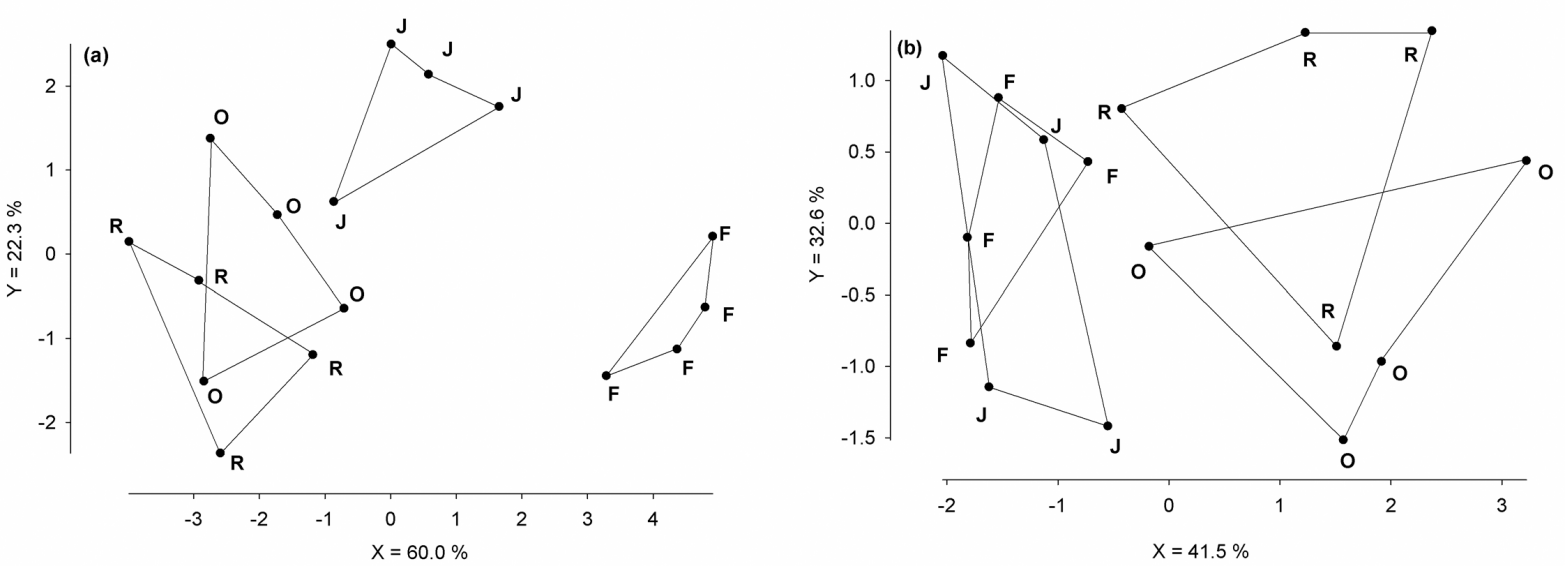
**Discriminant function analysis of living testate amoebae in litter (a) and soil (b) of four land-use systems:** rainforest (F), jungle rubber (J), rubber (R) and oil palm (O).

DFA separated the testate amoebae community composition in the litter of rainforest from that in litter of rubber (MD^2^ = 49.6***) and oil palm (MD^2^ = 41.3***). The communities in litter of rainforest and jungle rubber were also distinct by DFA, although less so (MD^2^ = 22.3**; Wilk’s λ 0.261, F_9,24_ = 9.41***; Fig 2A). DFA separated the testate amoebae community in rainforest soil from that in oil palm (MD^2^ = 10.0**) and that in rubber (MD^2^ = 7.28*), but not from that in jungle rubber (MD^2^ = 0.06^ns^; Wilk’s λ 0.216, F_9,24_ = 2.38*; Fig 2B).

**Fig 3:**
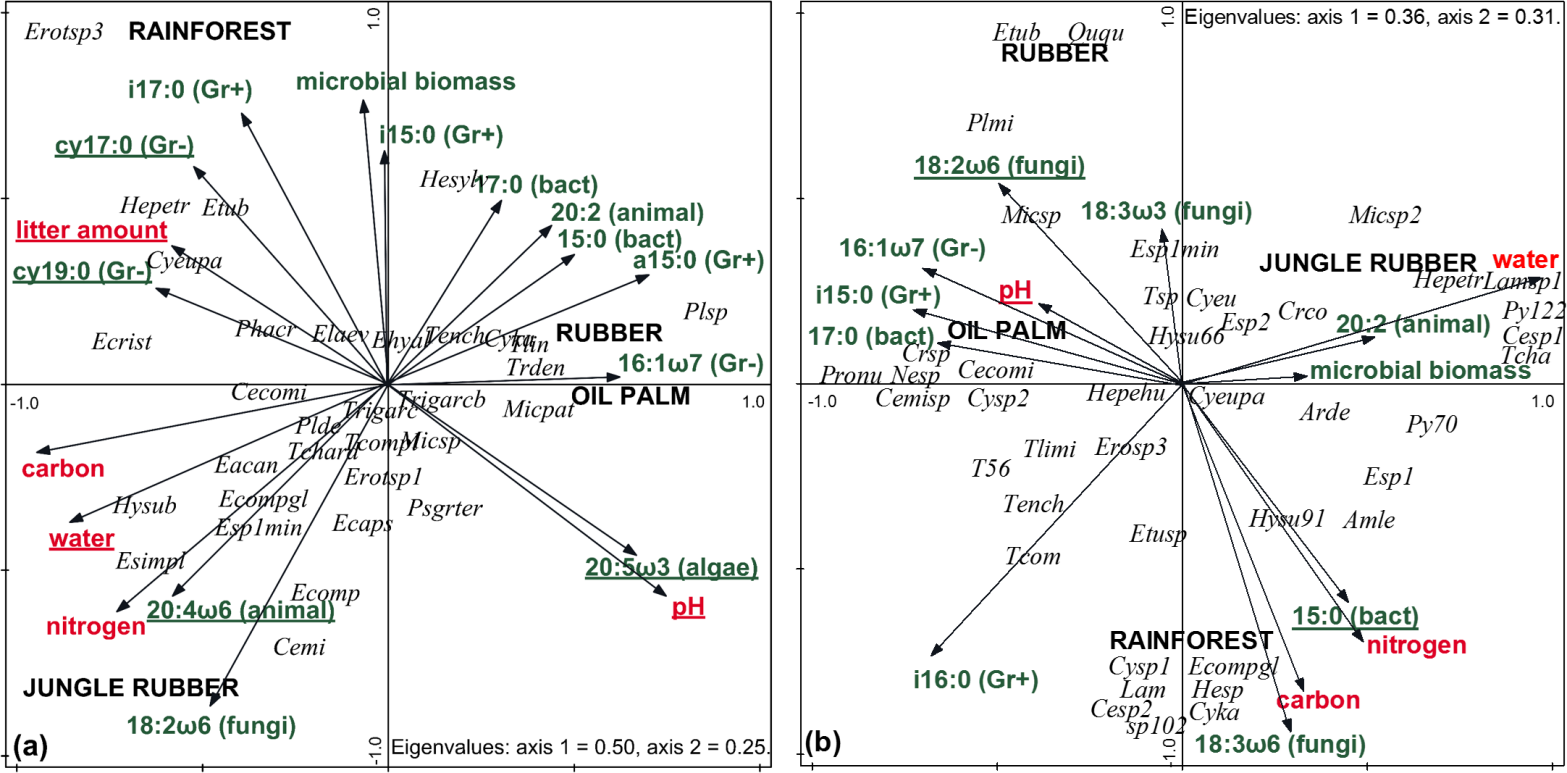
**CCA of living testate amoebae of litter (a) and soil (b).** Underlined factors are significant; abiotic factors marked in red, biotic factors marked in green; for full species names of testate amoebae see S2 Table; Bact, bacteria, Gr-, Gram-negative bacteria, Gr+, Gram-positive bacteria.

In CCA under forward selection, 7 of 18 litter environmental variables were significant as the first variable in the model (p<0.05; Fig 3A; S3 Table). Together, the 18 variables accounted for 82.7% of the variation in species data with the trace being significant (1.67, F = 2.38**). However, the first two axes explained most of the variation (axis 1 50.0% and axis 2 24.9%). Under forward selection of the environmental variables, pH accounted for the largest amount of variation in species data (14.8% of total; F = 4.5**). PLFA 20:5ω3 was the second environmental variable with significant explanatory power (accounting for an additional 8.3% of the variation (F = 2.9*), followed by PLFA 20:4ω6,9,12,15 (accounting for an additional 7.5%; F = 2.4*), amount of litter (7.4%; F = 2.7*), PLFA cy19:0 (5.6%; F = 2.2*), PLFA cy17:0 (4.8%; F = 2.0*) and water content (3.9%; F = 1.8*; see S3 Table). The remaining 34.7% of the variation was explained by variables with an explanatory power of less than 3%.

In CCA under forward selection, 3 of the 15 soil environmental variables were significant as the first variable in the model (p<0.05; Fig 3B; S3 Table). The variables included accounted for 60.7% of the variation in species data with the trace being significant (3.59, F = 1.24*). The first two axes explained 67% of the total variation (axis 1 36.2%, axis 2 31.2%). Including the explanatory variables one after the other using forward selection revealed that pH accounted for most of the variation in species data (7.5% of total; F = 2.0 **). The second environmental variable with significant explanatory power was PLFA 18:2ω6,9 accounting for an additional 7.2% of the variation (F = 1.9*), and the third was PLFA 15:0, accounting for another 6.8% of the variation (F = 1.9*; see S3 Table). The remaining 39.2% of the variation were explained by variables with an explanatory power of less than 3%.

### Trophic level

**Fig 4:**
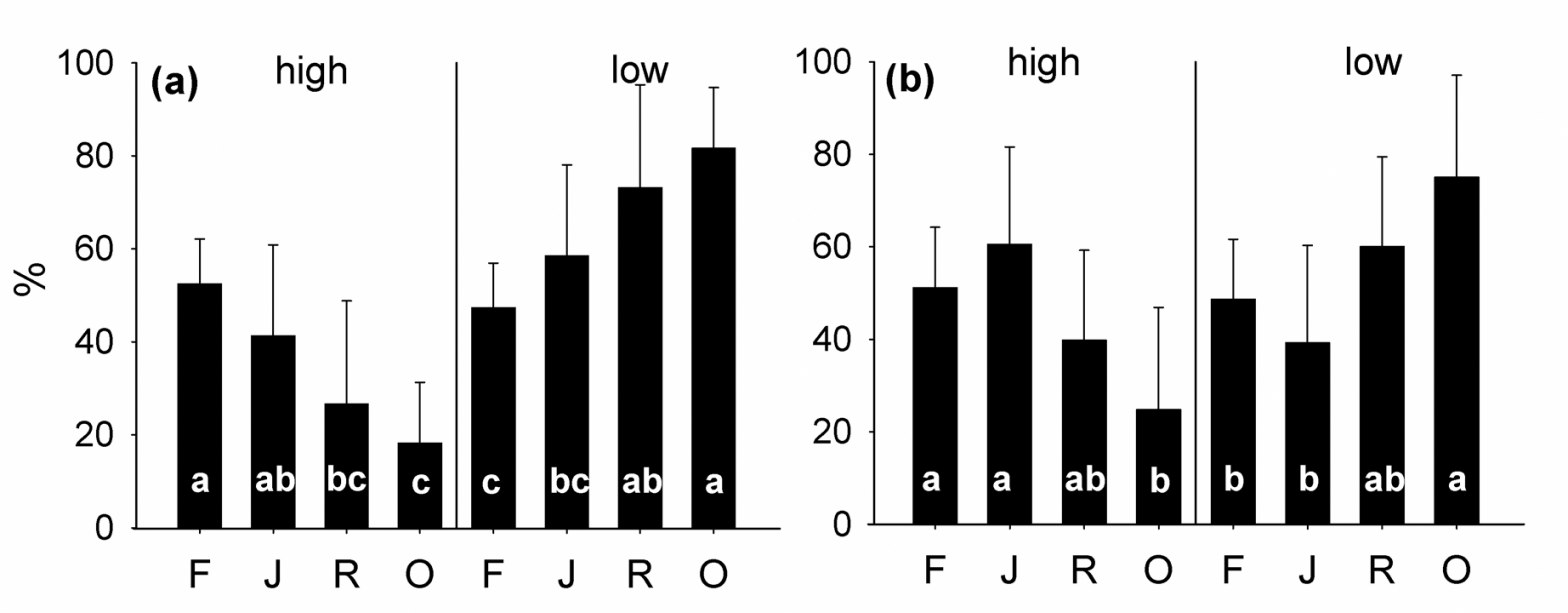
**Relative density (percentages of total) of testate amoebae species of high and low trophic level in litter (a) and soil (b) of four land-use systems:** rainforest (F), jungle rubber (J), rubber (R) and oil palm (O); mean with SD (n = 8). Bars sharing the same letter do not differ significantly (Tukey’s HSD test, p<0.05).

In litter the relative density of high and low trophic level species significantly differed between land-use systems (Wilk’s λ 0.584, F_3,28_ = 6.62***; Fig 4A). The relative density of high trophic level species was highest in rainforest, lowest in oil palm plantations and intermediate in jungle rubber and rubber with the opposite being true for low trophic level species. Overall, the trophic structure of testate amoebae in jungle rubber resembled that in rainforest and the trophic structure in rubber resembled that in oil palm. High trophic level species were negatively correlated with soil temperature (r = -0.59***) and pH (r = -0.52**), and positively with water content (r = 0.43*), amount of litter (r = 0.64***), PLFA i17:0 (Gr+; r = 0.41*), PLFA cy17:0 (Gr-; r = 0.39*), PLFA 20:4ω6,9,12,15 (animal; r = 0.52**) and PLFA 20:2 (animal; r = 0.38*). In contrast, low trophic level species were positively correlated with soil pH (r = 0.52**) and negatively with water content (r = -0.43*), amount of litter (r = -0.64***), PLFA i17:0 (Gr+; r = -0.41*), PLFA cy17:0 (Gr-; r = -0.39*), PLFA 20:4ω6,9,12,15 (animal; r = -0.52**); for the mean values see S3 Table.

As in litter, in soil the relative density of high and low trophic level species also differed significantly between land-uses (Wilk’s λ 0.635, F_3,27_ = 5.16**; Fig 4B). The relative density of high trophic level species was highest in jungle rubber and rainforest, lowest in oil palm and intermediate in rubber with the opposite being true for low trophic level species. High trophic level species were negatively correlated with total fungal marker (sum of PLFAs 18:2ω6,9, 18:3ω6, 18:3ω3; r = -0.45*) and with PLFA 16:1ω7 (Gr-; r = -0.44*), but positively with PLFA 20:2 (animal; r = 0.51**). Low trophic level species were negatively correlated with PLFA 20:2 (animal; r = -0.51**) and positively with total fungal marker (r = 0.45*) and PLFA 16:1ω7 (Gr-; r = -0.44*); for the mean values see S3 Table.

### Shell composition

In litter, the relative density of testate amoebae with siliceous shells significantly differed between land-use systems (Wilk’s λ 0.702, F_3,28_ = 4.0*). The relative density of species with siliceous shells was similar in rainforest (68.2±19.5% of total) and jungle rubber (69.8±16.4%) and significantly lower than in oil palm (33.7±17.3%) and rubber (30.2±19.3%). Species with siliceous shells were negatively correlated with soil temperature (r = -0.45*) and PLFA 20:2 (animal; r = -0.43*), and positively with water content (r = 0.49**), amount of litter (r = 0.44*), PLFA cy17:0 (Gr-; r = 0.46*) and PLFA 20:4ω6,9,12,15 (animal; r = 0.39*); for the mean values see S3 Table.

In soil, the relative density of testate amoebae with siliceous shells differed little between land-use systems; it was on average 49.7±19.7% (Wilk’s λ 0.970, F_3,27_ = 0.2^ns^).

## Discussion

### Structure of testate amoeba communities

In agreement with our first expectation, the impact of rainforest conversion on living testate amoebae increased with increasing management intensity. Macroinvertebrate diversity and plant diversity are similarly affected [33,38]. However, the opposite is true for prokaryote diversity which is higher in managed systems than in rainforests [58]. The order in which management intensity affected the community structure of testate amoebae was not what we expected. The order also differed for the different variables measured, species richness, density and biomass. The species richness of testate amoebae was similar in rainforest and jungle rubber, but lower in rubber and lowest in oil palm. In contrast, the density of testate amoebae decreased more continuously from rainforest to jungle rubber to oil palm to rubber, with the decline being more pronounced in soil than in litter. The biomass of testate amoebae was highest in rainforest, but in litter it reached a similar low level for each of the three land-use systems, whereas in soil it was similar in rainforest and jungle rubber, and lower than this in oil palm and lowest in rubber. Changes in species richness, density and biomass in testate amoebae with changes in land use were therefore more variable than in micro- and macroarthropods [33,38]. The oil palm plantations in our study had been fertilized in 2013 but the rubber plantations had not. Therefore nitrogen availability in oil palm presumably exceeded that in rubber [36]. This may well have contributed to the higher biomass and density of testate amoebae in oil palm compared to rubber plantations [16]. The beneficial effects of nitrogen fertilization are related to the increased availability of high quality food resources that benefit from nutrient additions, presumably bacteria and algae. Indeed, in oil palm plantations, bacteria flourished in soil and diatoms in litter (for details see Fig 3, S3 Table; [32,58]), whereas fungi, known to antagonistically affect testate amoebae [16,59], dominated in rubber plantations [32]. Furthermore, in line with our expectations, the reductions in density of testate amoebae were more pronounced in high than in low trophic level species (presumably feeding on other protists, nematodes, or small metazoan). In contrast, the density of low trophic level species (presumably feeding on bacteria and algae) increased in oil palm plantations. This shift in trophic groups may have been, at least in part, due to fertilization and associated disturbances.

The community composition of testate amoebae was also strongly affected by the conversion of rainforest into agricultural production systems in both litter and soil. In litter the community composition of testate amoebae of rainforest differed from that in each of the converted systems whereas, in soil, community composition in rainforest only differed from that in oil palm and rubber, but not from that in jungle rubber. This supports our second expectation that the impact of rainforest conversion on testate amoebae is more pronounced in litter than in soil. Of the 18 environmental variables of the litter layer seven were significant and explained 52% of the variation in species data with 26% explained by abiotic and 26% explained by biotic factors. In contrast, in soil only three of 15 environmental variables measured were significant explaining 21% of the variation in species data, with abiotic factors (7.5%; pH) explaining only about half of the variation explained by biotic factors (14%; bacterial and fungal PLFA markers). This suggests that, in litter, abiotic factors were more important in structuring testate amoebae communities than in soil. However, in litter and in soil testate amoebae are highly dependent on pH. In litter and soil of oil palm (5.8/4.6) and rubber plantations (5.3/4.3) pH was higher than in rainforests (4.3/3.8). The increase in pH was associated with an increase of Gram-positive bacteria, fungi and algae [32], whereas in rainforest Gram-negative bacteria were more abundant. This is understandable because Gram-negative bacteria are less sensitive to acidic conditions [60]. Thus, changes in the composition and structure of testate amoebae communities with land use were likely, in part, due to changes in microbial community composition induced by changes in soil pH [16,59,61]. Furthermore, the large amounts of litter in land-use systems other than rainforest and the great power of this factor in explaining the community structure of testate amoebae, suggest that conversion of tropical rainforest into oil palm and rubber plantations results in reduced habitat space for protists. Water content and land-use intensification significantly affected variations in species data in litter, but not in soil. This indicates that the impact of changes in abiotic conditions with rainforest conversion is less pronounced in soil than in litter, presumably due to the litter layer buffering fluctuations in abiotic factors [32].

### Trophic groups of testate amoebae

The relative density of testate amoebae of high trophic level in litter and soil decreased from rainforest (52%) to oil palm plantations (22%), i.e. with increasing management intensity, supporting our third expectation. This indicates that in rainforest the density of testate amoebae species of high and low trophic level are balanced and both are represented by a high number of functionally redundant species. In contrast, in other land uses, especially oil palm plantations, reductions in diversity are more pronounced in high trophic level species. This is probably associated with losses in the ability of predators to control prey populations, a pattern resembling that in macroarthropods [33]. These changes were notably less pronounced in soil than in litter, confirming our second expectation. In the litter layer, the density of high trophic level species was related to abiotic factors, such as temperature, pH and water content whereas, in soil, biotic factors were more important. This suggests that biotic coupling and top-down forces are more pronounced in the micro-food web of soil than in litter where abiotic forcing predominates.

### Testate amoebae with siliceous shells

The relative density of species with siliceous shells in litter was similar in rainforest and jungle rubber, but lower by more than 50% in rubber and oil palm, supporting our fourth expectation. This difference in density of testate amoebae with siliceous shells suggests that rainforest conversion affects the biogenic silicon pool and increases silicon losses. The results indicate that this was mostly due to higher soil temperature, lower water content, lower amounts of litter and lower density of certain bacterial groups in rubber and oil palm plantations (for details see S1 and S3 Tables and Shell composition section above). This again points to the importance of the litter layer in buffering the soil against environmental fluctuations [32]. Climate, system age and vegetation cover regulate protozoan silicon pools [27,34]. Moreover, testate amoebae are important consumers and suppliers of silicon, with the amount of silicon fixed by siliceous shelled testate amoebae in forested ecosystems being similar to that fixed by the trees [26,27]. Overall, our results show that land use essentially controls the biogenic silicon pool, as it does in the temperate zone [62]. Furthermore, the decrease in density of species with siliceous shells with management intensity suggests that these species may serve as indicators reflecting changes in the biogenic silicon cycle in converted ecosystems.

Overall, the results suggest that protist communities respond sensitively to the conversion of lowland rainforest into rubber and oil palm plantations. Conversion reduces species richness, density and biomass, and impairs important functional groups of soil microfauna. Conversion thus negatively affects the structure of the microfauna community and its functioning. The shift in trophic levels and losses of functionally redundant species may impact decomposition processes, nutrient mineralization and plant nutrient uptake. Changes in the biogenic silicon pool may also affect plants as silicon plays a major role in plant performance [31] and the carbon cycle [28,30]. Therefore, changes in testate amoebae community structure may allow a deeper understanding of changes in ecosystem functioning with changes in land use. As soil protists are major regulators of soil microorganisms and their functions, they need closer consideration if we are to understand changes in ecosystem functioning with changes in land use.

## Supporting Information

S1 Table Testate amoebae species list with mean density (a) and relative abundance (b) in the four land-use systems (F, rainforest; J, jungle rubber; R, rubber plantation; O, oil palm plantation) of the two localities studied (H, Harapan; B, Bukit Duabelas).
(XLSX)

S2 Table Characteristics of testate amoebae species (and abbreviations used in Fig 3) used for ascribing them to functional or trophic groups: shell composition, shell and aperture size, trophic level, sources for trophic level classification (see text for details). (XLSX)

S3 Table Means and standard deviation of environmental factors used in the study. (XLSX)

S4 Table Pearson correlations between environmental factors and density of testate amoebae species in litter and soil. (XLSX)
